# Community-based follow-up of very low birth weight neonates discharged from a regional hospital in Cape Town: a descriptive study

**DOI:** 10.1080/16549716.2025.2466277

**Published:** 2025-02-21

**Authors:** Thandi Maya Gondwana de Wit, Tanya Doherty, Michael Hendricks

**Affiliations:** a Department of Paediatrics and Child Health, University of Cape Town, Cape Town, South Africa; b Health Systems Research Unit, South African Medical Research Council, Parow, Cape Town, South Africa

**Keywords:** Neonatal morbidity and mortality, very low birth weight neonates, community health workers, community-based follow-up, community health services

## Abstract

**Background:**

Neonatal mortality remains a global health concern. In South Africa, 32% of under-five mortality consists of neonates, with 48% of neonatal deaths attributed to prematurity. The Home and Community-Based Services (HCBS) aim to reduce deaths of very low birth weight (VLBW) neonates through community health worker (CHW) home visits.

**Objectives:**

This study aimed to describe a cohort of VLBW neonates discharged from a regional hospital including their community follow-up, clinical outcomes and caregivers’ perceptions of the HCBS.

**Methods:**

This was a descriptive mixed methods study. Routine hospital health information from 1 January to 31 December 2018 was analysed to describe the cohort. The referral pathway and follow-up were assessed through stakeholder meetings and analysing referral forms. Caregivers were interviewed for HCBS data.

**Results:**

There were 169 VLBW neonates. The mean (SD) gestational age was 30 (±2.21) weeks, and the median (IQR) birthweight was 1210 g (1045–1390 g). At delivery, 85% had respiratory distress and 64% had presumed sepsis. Maternal characteristics included primigravida deliveries (15%), smoking (11%), alcohol use (9%) and teenage pregnancy (5%); 14% required social worker referral. Folder reviews showed referral plans for 49 (43.4%); however, 20 (17.7%) forms were received by HCBS. All five of the interviewed caregivers had positive perceptions of the HCBS.

**Conclusion:**

This study demonstrated a high burden of medically and socially vulnerable VLBW neonates discharged from a regional hospital. Even with established HCBS systems, few VLBW neonates were followed up at home. For the HCBS to be fully effective, promotion, strengthening and monitoring of the referral system are required.

## Paper Context

- **Main findings:** There is a high burden of medically and socially vulnerable very low birth weight neonates discharged from a regional hospital, and despite an established community-based follow-up programme, few of these neonates were followed up.
- **Added knowledge:** Several challenges both from the discharging hospital and the home and community-based service sides were identified, which can aid in the promotion, expansion and strengthening of this programme.
- **Global health impact for policy and action:** Globally, many health systems are using community-based services as integral members of the health team; however, these services need to be further researched and expanded upon to improve health outcomes.

## Background

Neonatal mortality continues to be a public health challenge in low- and middle-income countries (LMICs) where neonates have a 30-times higher risk of dying during the neonatal period (birth to 1 month of age) than infants during the post-neonatal period (29–365 days after birth) [1,2]. Despite the 47% reduction in the global neonatal mortality rate (NMR) from an estimated 5 million neonatal deaths in 1990 to 2.3 million in 2022, it is falling at a much slower rate than the mortality rate for children between 1 and 59 months [2–6]. There are discrepancies in the NMR between countries, with 99% of neonatal deaths occurring in LMICs, particularly in sub-Saharan Africa [2,6,7]. Like globally, neonatal deaths in South Africa (SA) are the main contributor (32%) to under-five mortality and have remained unchanged at 11–12 deaths per 1000 live births from 2012 to 2019 [5,8].

In 2021, prematurity was the leading cause of neonatal deaths globally at 37%, and in 2019, in SA, it was 36% [9,10]. Low birth weight (LBW), defined as a birth weight <2.5 kg, is an important risk factor for neonatal mortality, being associated with 60–80% of neonatal deaths [2,11,12].

A child death review (CDR) pilot study in SA revealed that lower respiratory tract infections (LRTIs) were the commonest cause of death, particularly in infants (52%), and that infants were more likely to die at home (69%) [13]. These deaths were significantly associated with prematurity and LBW and occurred soon after hospital discharge [13]. The CDR concluded that poor social and environmental factors, inadequate maternal support and lack of community-based services were major contributors to under-five home deaths, particularly in vulnerable preterm neonates [13]. Previous mortality reports focusing on health facility deaths found that 38% of stillbirths and neonatal deaths had modifiable family- or community-related factors [1].

Achieving the third Sustainable Developmental Goal (SDG) by 2030 requires a greater focus on neonatal mortality both in and out of hospital [3]. A post-natal package of care implemented with locally specific community-based interventions is imperative to reduce neonatal deaths [5–7,12].

The Home and Community-Based Services (HCBS) are an approach to decentralise health care by utilising community health workers (CHWs) to reach more of the population at a household level [7,14,15]. These CHWs have some basic training and work either as volunteers or on a basic salary [7,14,15]. CHWs are linked with the health system but work, in their communities, by providing education and promotive and preventative health care and, in some countries, the diagnosis and treatment of common conditions [7,14,16]. A large proportion of the HCBS focuses on maternal, child and neonatal health including vaccinations, breastfeeding, support and education [14].

The positive impact that community-based interventions have particularly on neonatal mortality has been emphasized in various global and local studies [7,11,12,14,17,18]. A Cochrane review found that community-based interventions reduced neonatal mortality by 25% [11]. A study in Nepal demonstrated that community-based visits resulted in mortality reduction from LBW with a relative risk of death being 84% less in those receiving community visits [12]. Community-based interventions also increased the caregiver’s health-seeking behaviour by 42% and breastfeeding rates by 93% [7,11,17]. By providing support, breastfeeding education, danger-sign recognition and early health-seeking education, CHWs could ensure that infants and children survive and thrive within vulnerable communities [12].

Since 2011, CHWs in SA have been part of the PHC re-engineering plan with emphasis on promotive and preventative health care [16,17]. In the Western Cape (WC), the HCBS is run by the WC Department of Health [16,18]. HCBS co-ordinators plan, monitor and evaluate the service in collaboration with non-profit organisations (NPOs) that employ nurse practitioners (NPs) and CHWs [16,18]. In 2016, there were 90 NPOs linked to the HCBS employing 3,600 CHWs with a provincial coverage of 0.77 CHW per 1000 population [16]. The CHWs worked 4.5 h a day at the time of the study (now they work 8 h a day), have undergone training in maternal and child health and are paid a basic salary [16,18]. The plan provincially is for one CHW to cover 250 households or 1000 people [16]. This ratio is challenging in providing adequate follow-up given the adult and child population they are required to cover [17].

Since 2015, guidance was established for the referral and follow-up of children from district and regional hospitals to HCBS in the Cape Town Metro District [19]. Children referred to HCBS include those with very low birth weight (VLBW) (i.e. a birthweight <1500 g), severe acute malnutrition and those on anti-retroviral medication referred for continued care at PHC services. An evaluation of the referral process and the utilisation of the HCBS have not previously been undertaken in the district. There are concerns that the service is underutilised. Therefore, the aim and purpose of the study were firstly to describe the characteristics (co-morbidities and socio-demographics) of the VLBW neonates born at a regional hospital in the Western sub-district of the Cape Metro over a year period. Secondly, it was to gain knowledge and understanding of the referral process to HCBS, the follow-up of these neonates by CHWs, their outcomes and the caregivers’ perceptions of the service with the aim of strengthening this referral pathway and service.

## Methods

The study constituted the research requirement for a specialisation MMed degree in Paediatrics by the first author [20].

### Study design

This was a descriptive mixed methods (including both quantitative and qualitative data) study of a VLBW cohort following hospital discharge over a 1-year period between 1 January and 31 December 2018. It used quantitative data from an accredited hospital database, patient folders and referral forms. The qualitative data were obtained from meetings with NPOs and semi-structured caregiver interviews.

### Ethical approval

Approval for the study was obtained from the Departmental Research Committee, School of Child and Adolescent Health as well as the Human Research Ethics Committee of the Faculty of Health Sciences, University of Cape Town (HREC Ref 582/2019)

### Study setting

The Cape Town Metro District of the WC Province is situated on the southern peninsula of the province and is divided into eight sub-districts (Supplementary file 1) [21]. The regional hospital where the study was based is situated in Cape Town and serves the Western sub-district population [21]. Within this sub-district are two NPOs who employ seven professional nurses and three enrolled nurses to coordinate 192 CHWs covering a population of 60 000 [22].

### Study participants

Only the VLBW neonates discharged from the hospital during the study period were included in the study population. Neonates excluded from the study included those with a birthweight >1.5 kg who are not included in the HCBS service, all in-hospital deaths and those whose discharge summaries or folders were unavailable. The VLBW neonates living in the Western sub-district formed the study cohort, and their caregivers were eligible for the interviews. Further exclusions from the study cohort included transfers to other hospitals (for either step-up or step-down hospital care), other hospital deaths or those discharged to other sub-districts and not followed up by the sub-district HCBS team. The HCBS referral forms found in the study cohort’s folders and those received by the HCBS were analysed (Supplementary file 2). Caregivers whose neonates died or required transfer were also excluded from interviews. Only VLBW neonates where a referral form was received by the HCBS sub-district coordinator were in the sub-cohort of caregivers who were contacted for interviews.

### Data collection methods

Discharge records from the regional hospital were used to identify the VLBW study population and the study cohort. Quantitative data were extracted from an accredited hospital database, the Electronic Continuity of Care Record (ECCR) [23]. Patient records were obtained and analysed when information on ECCR was incomplete or unavailable. These data aided in describing the burden of VLBW neonates (gestational age, birthweight, co-morbidities and potential social risk factors) and to identify the HCBS referral statistics. There were missing data for certain outcomes where the information was not recorded in the records or included in ECCR. We have indicated this in the tables by stating the number of participants for which information was ‘not recorded’.

Meetings were held with the hospital staff, the sub-district co-ordinators and a nurse practitioner (NP) from one of the NPOs to obtain information regarding the referral pathway and follow-up of the VLBW neonates.

The referral forms received by the HCBS or found in the patient’s folder were used to assess completion of the form by the referring facility and information regarding follow-up required.

The sub-cohort of participants invited for interviews was those caregivers of VLBW neonates whose referral forms were received by the HCBS sub-district co-ordinator and followed up. Given the COVID-19 global pandemic, the interviews changed from face-to-face to telephonic interviews in November 2020. The questionnaires included data on the caregivers’ socio-demographic status, the growth, immunisation status, feeding and hospital admissions of the neonates and parental health education. Information related to the HCBS follow-up included the number of visits, health topics covered and caregivers’ perceptions of the utility of the service and suggestions for improvement (Supplementary file 3). The questionnaires were translated from English into Afrikaans and isiXhosa and back translated. Prior to the study, the questionnaires were piloted and revised. The first author with the assistance of a trained interpreter administered the questionnaires. The interviews were conducted in the language preferred by the caregiver, and given that they were telephonic, verbal consent from the caregiver was obtained because paticipants were unable to submit electronic consent forms. The interviews were recorded and transcribed.

### Data analysis

The quantitative data regarding the VLBW population and cohort were extracted into an Excel 2013 (Microsoft, USA) spreadsheet, and simple descriptive analyses were done using Stata 2015 (StataCorp., USA) [24]. Summary statistics included frequencies, percentages, means with standard deviations for parametric data and medians with interquartile ranges for non-parametric data. No group comparisons were made, and thus, inferential statistics were not performed. The first author performed the analyses. The primary outcomes related to the study cohort were the number discharged from hospital and those that demised as well as those with specific co-morbidities. The outcomes related to the sub-cohort were whether they had been followed up by the community health worker, if they were still alive and any medical conditions or health concerns the child had developed post-discharge. For the analysis of the qualitative data, the first author and independent researchers listened to the recordings and read the transcriptions to gain familiarity with the data. A content analysis approach was applied using predominantly deductive coding according to the focus of the open-ended interview questions [25]. The data were managed in MS word software and categorised into themes with illustrative quotes.

## Results

### Study population, cohort and sub-cohort

In 2018, 1172 neonates were discharged from the regional hospital, of which 169 were VLBW, making up the study population. The VLBW neonates constituted 13.2% of the discharges to home, 29.3% of transfers and 57.9% of the in-hospital deaths.

**Figure 1.**
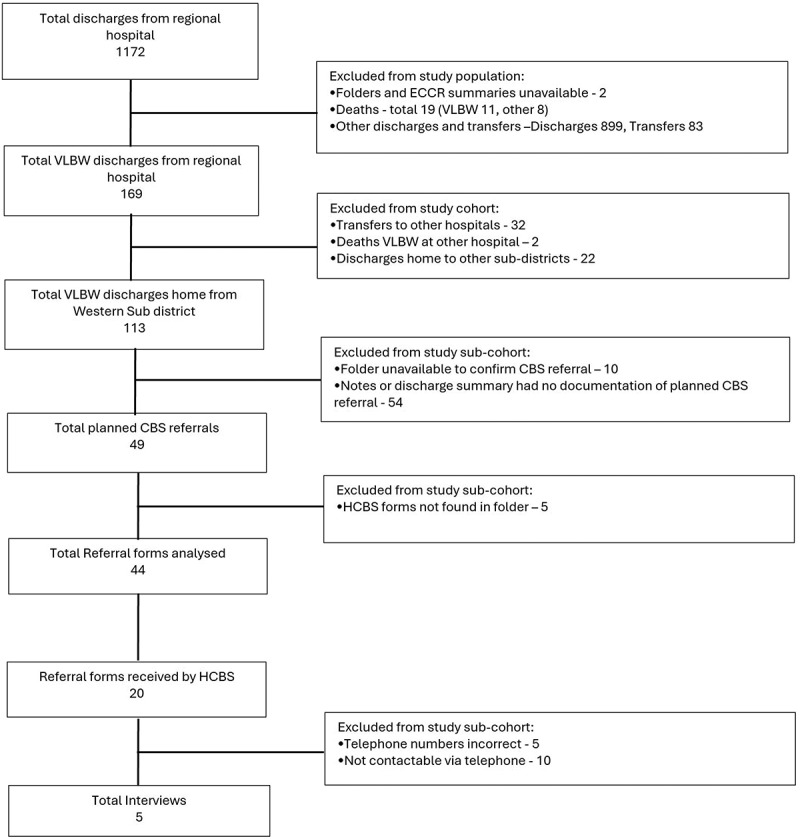
Flow diagram depicting the inclusion and exclusion of VLBW neonates for follow-up.

Figure 1 depicts the determination of the VLBW study cohort and the study sub-cohort for follow-up. The 169 neonates forming the overall study population were analysed for general birth information, co-morbidities and social factors. One hundred and thirteen VLBW neonates (67%) were included in the study cohort as they were from the Western sub-district where the NPOs functioned. Only 49 (43.4%) of the discharged neonates had any documentation of the HCBS referral, and of these, only 44 (38.9%) forms were found for analysis. HCBS only received 20 (17.7%) referrals during the 1-year period, which constituted the study sub-cohort that was eligible for interviews. However, a total of five caregivers could be located for an interview (Figure 1). The other caregivers could not be contacted due to incorrect telephone numbers, telephone numbers no longer in use or no telephonic response.

### Birth indicators, comorbidities and social factors

The birth indicators of the study population are outlined in Table 1. The mean gestational age at delivery was 30 weeks, the median birthweight was 1210 g and hospitalisation was required for a median of 38 days. Of these neonates, 52.7% were delivered by caesarean section (C/S) and the major indications being a pathological cardiotocogram (CTG) (67%), of which 22% were for pre-eclampsia. In total, 35% of the mothers undergoing C/S suffered from pre-eclampsia.

**Table 1.** VLBW birth indicators (*n* = 169).

Variable	Outcome
**Delivery gestational age**	
Mean (SD) gestation at delivery Number (%) of extreme premature neonates (<28 weeks)	30 (± 2.21) weeks 20 (11.8%)
**Delivery weight**	
Median (IQR) weight (g) at delivery Number (%) of neonates <1000 g (ELBW) Number (%) of neonates with birth weight 1000–1500 g	1210g (1045-1390g) 33 (19.5%) 136 (80.4%)
**Hospital length of stay**	
Median (IQR) length of stay in hospital	38 (29–51) days
**Mode of delivery**	
Total number (%) NVD Total number (%) C/S Delivery mode not recorded	77 (45.6%) 89 (52.7%) 3 (1.8%)

SD – Standard deviation, % – percentage, IQR – Inter-quartile range, g – grams, ELBW – Extremely low birth weight.

Table 2 depicts the co-morbidities of the VLBW neonates. A large proportion of VLBW neonates (84.6%) had some form of respiratory distress with the majority requiring continuous positive airway pressure (CPAP) (59.8%). Thirty-two percent of the mothers of these neonates had not been given a dose of steroids prior to delivery. Sixty-four percent of VLBW neonates were managed at delivery for presumed sepsis, having two or more septic risk factors according to the hospital neonatal guidelines. Of these, 8.3% required 5 days of antibiotics due to high septic markers and only one neonate had culture-proven sepsis at delivery. Thirty-seven percent of the VLBW neonates were managed as possible nosocomial sepsis based on clinical signs, but only 4.1% had culture-proven nosocomial infections. Three neonates’ blood cultures grew *E. coli*, one *Streptococcus viridans*, one *Listeria*, one *Klebsiella pneumonia* and one *E. faecium*. A quarter of VLBW neonates were human immunodeficiency virus (HIV) exposed, with one neonate having a positive HIV polymerase chain reaction (PCR) test at birth. There were no tuberculosis (TB) exposed neonates in this cohort.

**Table 2.** VLBW neonatal co-morbidities (n = 169).

Co-morbidities	Number (n = 169)	Percentage (%)
**Respiratory distress at birth**	143	84.6%
**Maternal steroids received prior to delivery**		
None 1 dose 2 doses Not recorded	54 39 57 19	32% 23.1% 33.7% 11.2%
**Respiratory support at delivery**		
No respiratory support required Respiratory support required NPO2 HFNC CPAP IPPV/HFOV	31 38 7 17 101 13	18.3% 81.7% 4.1% 10.1% 59.8% 7.7%
**Sepsis at birth**		
Presumed sepsis at birth (2 or more septic risk factors present) Possible (raised septic markers at 48 h) Proven (culture confirmed infection)	108 14 1	64% 8.3% 0.6%
**Nosocomial sepsis**		
Presumed nosocomial sepsis (clinical deterioration in hospital) Possible (raised septic markers at 48 h) Proven (culture confirmed infection)	63 19 7	37.2% 11.2% 4.1%
**HIV exposed**	42	24.8%

NPO2 = nasal prong oxygen, HFNC = high-flow nasal cannula, CPAP = continuous positive airway pressure, IPPV = intermittent positive pressure ventilation, HFOV = high-frequency oscillatory ventilation, HIV = human immunodeficiency virus.

Nearly 10% of all VLBW deliveries included mothers with unbooked pregnancies (no antenatal care) (Table 3). Almost 15% were primigravida deliveries with nearly 5% of births involving mothers less than 18 years of age. The mean age of the mothers was 29 years (SD = 6.2). Eleven percent of pregnancies were associated with maternal smoking, nine percent with maternal alcohol abuse and three percent with maternal drug addiction. Fourteen percent of neonates were referred for social worker review (excluding those for teenage pregnancy) for reasons including poor socio-economic status, abandonment and poor maternal medication compliance.

**Table 3.** VLBW neonatal social factors (*n* = 169).

Social factors	Number (n)	Percentage (%)	Number not recorded (percentage)
Unbooked pregnancy	16	9.5%	9 (5.3%)
Neonates born before arrival (BBA)	7	4.1%	–
Primigravida pregnancies	25	14.7%	10 (5.9%)
Teenage mothers	8	4.7%	11 (6.5%)
Maternal smoking exposure	20	11.8%	39 (23.1%)
Maternal alcohol exposure	15	8.9%	36 (21.3%)
Maternal drug addiction exposure	5	3.0%	4 (2.4%)
Required social worker referral	23	13.6%	–

On discharge, 86% of the neonates were successfully breastfeeding. Seventy-nine percent were discharged home, while others were transferred to higher levels of care (4.8%) or to step-down hospitals to continue care (14.2%).

### The referral pathway and follow-up

The information regarding the referral pathway was obtained through meetings with the regional hospital staff, the Western sub-district’s HCBS co-ordinator and the NP of one of the NPOs and is summarised in Supplementary file 4.

All discharged VLBW neonates should be referred to the HCBS co-ordinators of the Western sub-district by the discharging doctor. The doctor is responsible for completing the form, providing the patient’s contact details and reason for referral; the caregiver is required to sign the form and grant permission for the home visit. More recent forms are pre-filled and designed to ease completion of the form (Supplementary file 2). Once the neonate is discharged, the forms are faxed by the ward clerk to the HCBS co-ordinators.

Once received, the HCBS co-ordinator distributes the referrals according to the residential area to the specific NP of the various NPOs. The NP then identifies a specific CHW to conduct the home visit. These CHW’s job descriptions are broad and not only include these VLBW neonatal follow-ups but also other paediatric and adult health promotive, preventative and rehabilitative services. Currently, there is no formal follow-up plan that includes the frequency and duration of visits. The follow-up plan is put together by the nurse practitioners after the CHW undertakes the initial home visit. The NP assists the CHW in planning the home visit for each patient. The CHW should visit the home of the VLBW neonate within a few days of discharge.

After the home visits, feedback sessions take place between NP and CHW. There is no standardised follow-up plan. Each neonate’s follow-up visit is individualised, discussed and monitored. Discharge from the HCBS is decided by the NP and CHW.

### Completion of the HCBS forms

Of the 44 hCBS forms found for analysis, most sections were adequately completed. However, only 84% of the forms had contact telephone numbers listed. The main reasons for referral of the neonates, in addition to being VLBW, included breastfeeding and nutrition support (89%), newborn care (55%), growth issues (34%) and immunisations (38%). Of the forms, 88.6% were signed by the caregiver granting permission for the CHW to visit their home. Seventy-five percent of the forms were the pre-filled referral forms which are pre-populated forms with follow-up requests for all VLBW neonates to ensure more efficient and easy referral of neonates.

### Follow-up home visits by CHWs

Of the 20 referrals received by HCBS, only five caregivers could be traced and interviewed. All caregivers were female and the biological mothers of the VLBW neonate, with four having other children at home. Only one mother was currently the daytime caregiver of the child with all other mothers having full-time jobs with their babies being cared for by an older child of 18 years who was out of school (1), a grandmother (1) or a nanny (2). Three of the interviewees were foreign nationals, and none of the South African mothers had a Child Support Grant. Supplementary file 6 depicts the demographics of the sub-cohort.

Three of the interviewees recalled being told about the home visit following discharge and giving consent for the visit. One mother was very concerned about the CHW visit as she was unaware of it.

I was also surprised … I didn’t even know they do check-ups and the whole system. I didn’t want to open the door … then they showed (their) ID. *(Mother 1)*

The occurrence of the initial visits by CHWs varied with one happening within a week of discharge, one within 2 weeks and one occurring more than a month post-discharge. The number and frequency of the visits varied for each neonate, but all were visited by the CHWs one to five times following discharge.

Three mothers were counselled on breastfeeding; however, the other health topics covered during the CHW visits varied.

They teach me how to breastfeed, they told me I must go for check-ups, the baby, I must do kangaroo. *(Mother 2)*

One mother recalled the CHW discussing how to make and use oral rehydration solution for gastroenteritis, one recalled discussing the importance of immunisations and two recalled discussing Kangaroo Mother Care and emphasised the importance of the Road to Health Book (RTHB), but none covered the danger signs and when to seek care. In addition to the health topics discussed, it was felt that information about how to care for a premature neonate would be useful.

### Outcomes and caregiver feedback

All those visited by the CHWs scored the CHW visits as either very or extremely useful on a sliding scale. The two other mothers, who did not have a follow-up, felt that they would have benefitted from a visit by a CHW.

Mother 3 said that she would have liked to have known, *‘how I must take care of such small baby’*.

None of the mothers had ever contacted the CHWs for help with health issues and felt if their child was sick, they would not contact them with questions as most would *‘take them* (i.e. their children) *to the clinic’.*

Two mothers elaborated saying that they did not know who the CHWs are or where to find them if the need arose and one said she would have liked to have contacted the CHW.

Of those interviewed, there was one child who died of gastroenteritis at the age of 9 months. This child had been living with the grandmother as the mother had to return to work after a month of maternity leave. The other four children were reported to be doing well. Only one child had been admitted to hospital for 2 days for a lower respiratory infection. One child was being followed up at the neurodevelopmental clinic at the regional hospital. All reported to still be in possession of the RTHB, and all children had received their immunisations. Three mothers breastfed their children for more than 6 months. The other two stopped at 1 month and 3 months, respectively, to return to work.

Of the four surviving children, each mother shared some ongoing stress, worry and concern about their babies. Three of the mothers had worries about the weight and feeding of their children, and one had concerns about the child not being able to stand.

## Discussion

The study identified a large burden of VLBW neonates with several medical and social factors, placing them at a high risk of morbidity and mortality. A review of the folders showed plans for HCBS referral in less than half of the cohort. However, the HCBS received only 17% of the referrals of the VLBW neonates discharged home. Despite the small sample size, community health worker visits were positively received and deemed beneficial by the interviewed mothers, highlighting the necessity of this service. This study also identified challenges in follow-up in the community from both the hospital and the HCBS perspective. However, further research into reasons for the challenges is required.

Of the VLBW neonates in this study, almost 20% and 10% had extreme low birth weight (ELBW) and extreme prematurity (<28 weeks), respectively. These neonates are at high risk of mortality and morbidity including long-term health and neurodevelopmental issues [26]. Most of them had respiratory distress at delivery requiring support with many remaining at risk of acute respiratory infection (ARI) following discharge. A South African birth cohort showed that in cases of community acquired pneumonia, 19% and 21% were premature and low birth weight neonates, respectively [27]. Sepsis can be difficult to diagnose during the neonatal period due to non-specific signs, and therefore, neonates in this study were treated whilst awaiting their results. Despite 64% of neonates at delivery and 37% during hospital admission developing signs of sepsis, only a small percentage had possible or proven infection. The Child Death Review group reported that a large proportion of premature neonates dies shortly after being discharged [13], highlighting the importance of early identification of infection both in-hospital and by caregivers following discharge. The benefits of breastfeeding are well known, and the data in this study were positive with three out of five caregivers electing to exclusively breastfeed for 6 months. Given the vulnerability of these neonates to ARI and sepsis, CHWs can play a vital role in promoting clinic attendance for immunisations, promotion and support of breastfeeding and counselling caregivers about danger signs and early health-seeking behaviour.

The study population highlighted numerous social problems. Overall, 14% of caregivers required social worker assistance and about one in five had the additional stress of being a first-time mother. VLBW or premature neonates are known to cause further stress for the parents with a study reporting 40–50% of them suffering from postpartum anxiety and depression [26]. This again highlights the essential role CHWs can play in the counselling and supporting these mothers following hospital discharge. Three of those interviewed were foreign nationals who do not qualify for a social support grant, which economically strains the family. It is concerning that none of the South African caregivers had a child support grant, which has been shown to be an important source of income for poor families [28].

The effectiveness of HCBS follow-up care for very low birth weight neonates (VLBW) relies on the successful completion of all steps in the referral pathway. Several constraining factors were identified in the study. Despite being a requirement, less than half of the VLBW neonates had a planned HCBS referral. Exploring the reasons for the lack of referral was beyond the scope of this study. However, some plausible reasons were deduced. There are many rotating junior and senior staff in the hospital’s neonatal department, which may influence compliance with the discharge package of care for these neonates. Poor record keeping may be another reason why there were plans to refer but no corresponding documentation. The referral forms found in the hospital folders indicate the likelihood that these were never handed to the clerk responsible for faxing them. Other possible reasons for the limited referrals could be the lack of a monitoring system, no relationship with HCBS, no feedback information loop, it mainly being a paper-based system where the loss of forms may occur and staff not appreciating the vital role that CHWs play in the follow-up and support of these neonates. Educating health professionals on the referral criteria as well as the process of referral not only ensures that VLBW and other groups are referred to the HCBS but also promotes this service and its increased utilisation and expansion. The use of electronic referrals via e-mail or through the electronic discharge system may be ways of ensuring that VLBW neonates are referred and their referrals received by the correct HCBS team. An electronic system is currently being piloted in another subdistrict; however, the outcomes are not available as yet.

Overall, completion of the forms by staff was good (Supplementary file 5). However, key contact information such as telephone numbers was not documented by the discharging doctor in nearly a fifth of referrals. The relevance of this information needs to be viewed in relation to the inability to undertake most of the interviews due to incorrect telephone numbers. Allocating responsibility for accurate form completion to one of the clerical staff could assist with documenting multiple contact numbers and correct residential addresses. These are essential to ensure the CHW visit and make vital contact with the family.

The CHWs who visit the families are employed by different NPOs. Currently, some CHWs have additional training in maternal and child health, but this is not standardised. In a CHW study in South Africa, CHWs expressed feeling that they lacked knowledge and skills and suggested further training and ongoing supervision being beneficial [18]. Given that there is no set plan or procedure for visits, it is challenging to compare the visits between caregivers. It was difficult to determine the frequency of visits performed by CHWs and the discharge criteria as no formal record was kept of the visits. Currently, the nurse practitioner meets regularly with the CHWs to identify issues raised during visits so that they can be dealt with swiftly and the HBCS co-coordinators also meet with the nurse practitioner to ensure smooth and efficient functioning of the service and feedback of information. The WHO has recommendations for a postnatal package of care which has been included in a Western Cape postnatal checklist [29]. This could be adapted to develop a post-discharge package of care which CHWs could use for the follow-up of VLBW neonates, thereby standardising the follow-up care and ensuring that specific health topics are addressed. This could also assist in training CHWs. This standardisation could also allow for the development of a procedural plan for contacting and following up of the referred patients. It would be advisable that all HCBS co-ordinators, CHWs and doctors are involved in planning for these VLBW follow-up visits.

There needs to be an improvement in linkages between the nearby primary health facilities, the referral hospitals and the HCBS to facilitate the success of this programme. A systematic review of CHWs highlighted the essential role that integration and collaborative relationships within the health system play in strengthening these programmes [14]. A Ugandan study assessing newborn home visits also highlighted the importance of these community linkages with health facilities in improving neonatal outcomes [30]. The discussion of caring for a VLBW neonate needs to start in-hospital and continue into the CHW visits. Developing a feedback loop between health professionals at the referring hospitals and the HCBS coordinators would be very beneficial to encourage accountability as currently there is no formal system in place. This would help ensure that all children referred are seen at follow-up, and issues identified by the CHW or the hospital can be easily fed back to the other. It is also vital to raise awareness within communities about CHWs, their role and how to contact them. Mothers in a South African CHW study mentioned the benefits of knowing they could quickly contact a CHW if there was a problem and that the CHW understood the needs of the mothers in their community [18]. This prior study also noted the appreciation for CHW visits as was found in our study [18].

Overall, there is limited data about the effectiveness of HCBS and other community interventions, and therefore, more quantitative and qualitative research into the HCBS needs to be undertaken. Further research should also include the perspective of the CHW and the HCBS to ensure that all aspects of the service are assessed and problems addressed.

### Strengths and limitations of the study

This study is the first to evaluate the referral service for VLBW neonates discharged from a hospital and receiving follow-up care from Home and Community-Based Services (HCBS) in the province. It has provided valuable information that can be used to strengthen the service. However, the study had several limitations including the low number of caregivers who could be traced and interviewed. This study was conducted early in the history of the HCBS at a regional hospital. The number of HCBS referrals related to existing VLBW neonates may differ from the 2018 cohort as the forms are now handed directly to the clerk for faxing and kept in a file for record keeping. There are anecdotal reports that following this study, there was an increase in referrals to HCBS post-promotional drives, but this requires further research. The fewer numbers of referrals from 2018 resulted in smaller study numbers for the interviews, which did not enable theoretical saturation and may impact the study conclusions. The study time period was 2018, but interviews took place in 2020 due to the first author completing her paediatric speciality and only having the time to undertake interviews during certain clinical rotations and delays caused by the ethics application process. The time lag may have contributed to many participants being uncontactable due to incorrect contact numbers or changing addresses, resulting in a loss of follow-up. This is a retrospective study, and therefore, there was a risk of recall bias occurring during the interviews. Due to COVID-19, the interviews were changed to telephonic interviews. The child’s RTHBs could not be analysed, and therefore, some outcomes could not be assessed. Home visits could not be performed. These might have resulted in more participants if the caregiver’s home address was unchanged since discharge, despite a change in phone number. The findings may not fully reflect the experiences of all mothers due to the interviewees being older as well as educated. Furthermore, we were not able to interview the daytime caregivers of the children who may have had different perspectives of CHW visits. Given the study timeframe, interviews with the NPs or CHWs were not able to be conducted and included in the study. However, it is felt that this would be a valuable addition to the study, and therefore, it is recommended that further qualitative research is done in this area.

## Conclusion

This study showed that there is a high burden of medically and socially vulnerable neonates who are discharged from a regional hospital in the WC, South Africa. Few neonates were followed up despite an established referral system. Even with several challenges in the HCBS system, the caregivers who did receive the service were positive about the HCBS, which provided health education, counselling, and support following hospital discharge. Therefore, in SA, this essential and valuable service needs further promotion, expansion and a strengthening of the referral system from healthcare facilities to reach all those in need and to have the desired impact in communities.

## Supplementary Material

Supplementary_figure_2.jpg

Supplementary_documents_for_manuscript.docx

Supplementary_figure_1.jpg

Supplementary_figure_4.jpg

## Data Availability

The datasets collected and analysed during this study are not publicly available due to patient contact details in the datasets and therefore the need to protect patient information. They are available from the corresponding author on reasonable request.
