# Supplementary figures and images for: Community-based follow-up of very low birth weight neonates discharged from a regional hospital in Cape Town: a descriptive study
Source: Glob Health Action. 2025 Feb 21;18(1):2466277. doi: 10.1080/16549716.2025.2466277 (PMC11849016; doi:10.1080/16549716.2025.2466277)

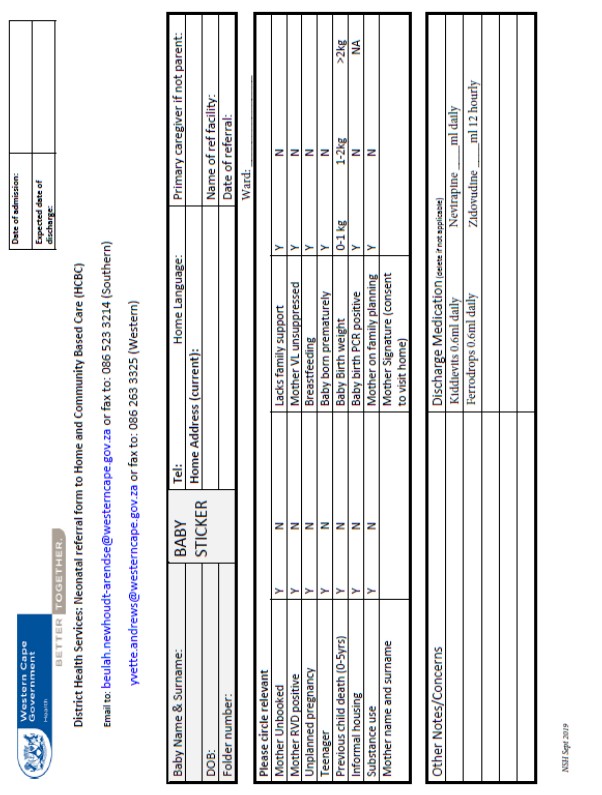

Supplement: Supplementary_figure_2.jpg [file ZGHA_A_2466277_SM0671.jpg]

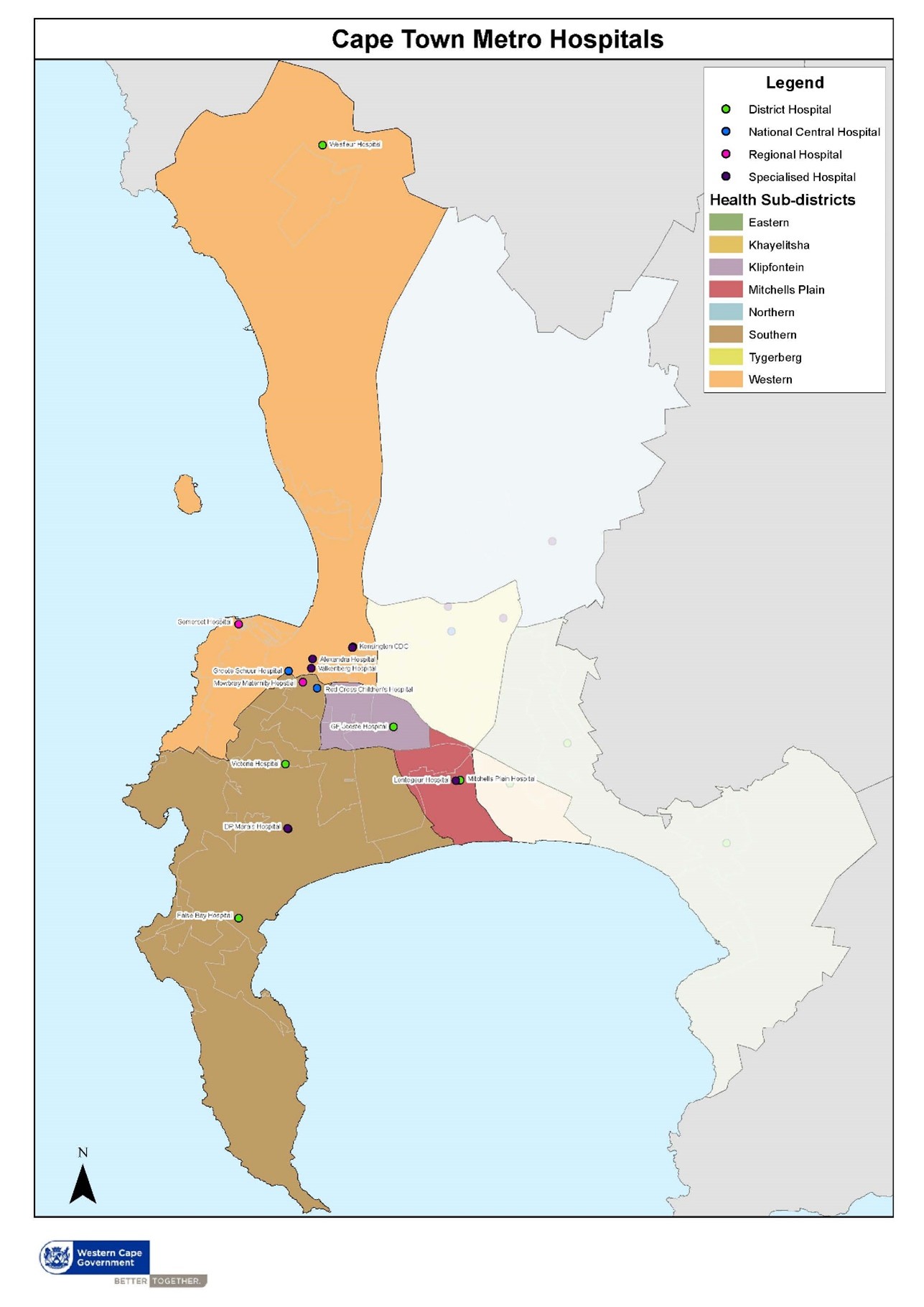

Supplement: Supplementary_figure_1.jpg [file ZGHA_A_2466277_SM0669.jpg]

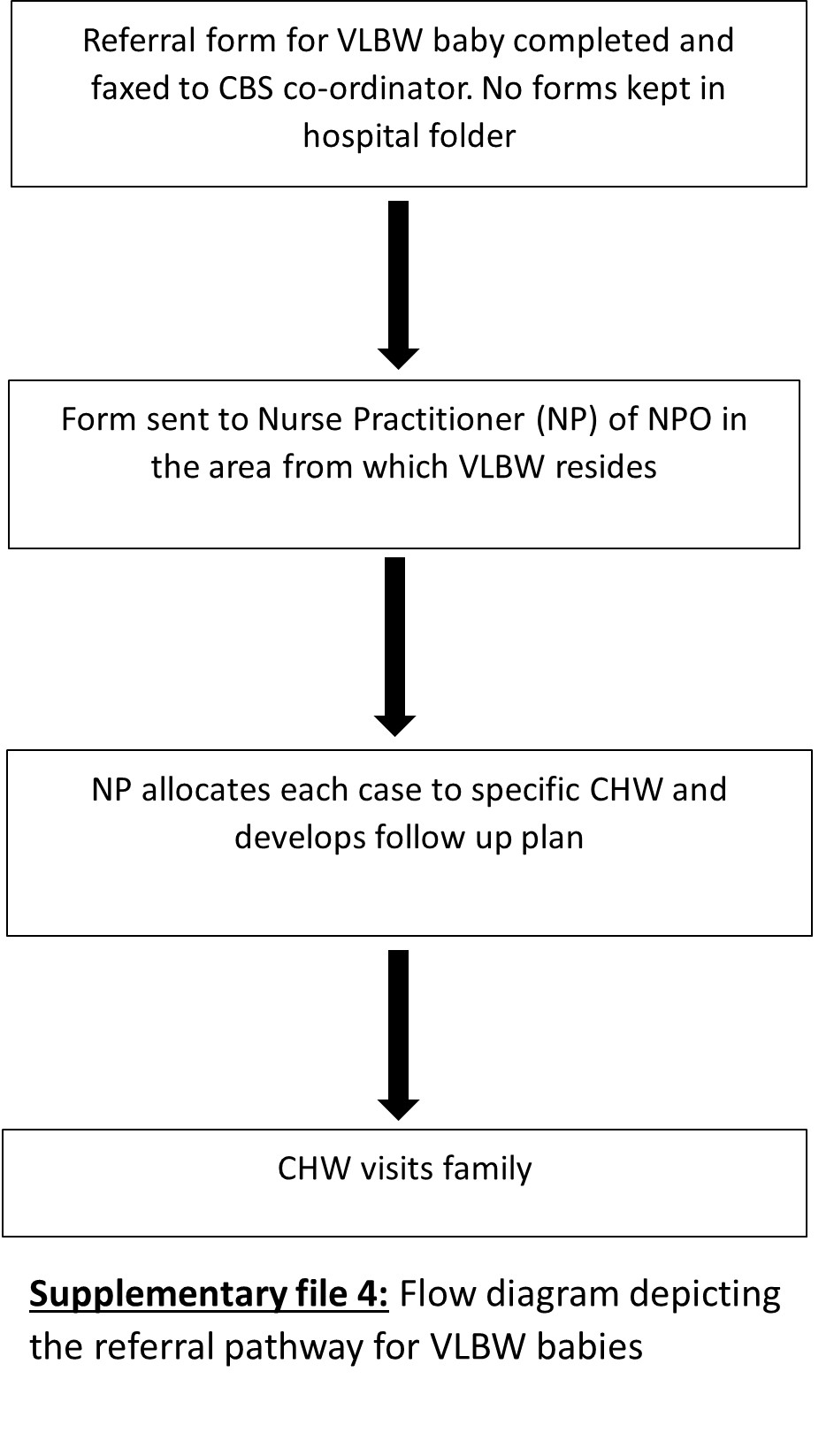

Supplement: Supplementary_figure_4.jpg [file ZGHA_A_2466277_SM0668.jpg]
