# Supplementary material for: Community-based follow-up of very low birth weight neonates discharged from a regional hospital in Cape Town: a descriptive study
Source: Glob Health Action. 2025 Feb 21;18(1):2466277. doi: 10.1080/16549716.2025.2466277 (PMC11849016; doi:10.1080/16549716.2025.2466277)
Supplement: Supplementary_documents_for_manuscript.docx [file ZGHA_A_2466277_SM0670.docx]

**Supplementary Files**

1 Map of Health Sub-districts of Cape Town Metro District of Western Cape

2 Pre-filled HCBS VLBW referral forms

3 Questionnaire

4 Flow diagram depicting the referral pathway for VLBW babies

5 HCBS referral form analysis (n=44)

6 Demographic characteristics of the study sub-cohort involved in the interviews

**Supplementary file 1:** Map of Health Sub-districts of Cape Town Metro District of Western Cape

**Supplementary file 2:** Pre-filled HCBS VLBW referral forms

**Supplementary file 3:** Questionnaire


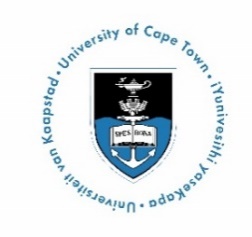


University of Cape Town

Department of Paediatrics

**MMed Research: Questionnaire**

**Research Study Title:** **A descriptive study of the community-based follow-up and outcomes of very low birth weight babies discharged from a regional hospital**

Thank you for completing the questionnaire. Please write answers in the provided spaces. Where to select an answer, please tick the appropriate answers.

Study number: ____________________________

Date completed (dd/mm/yyyy ):_________________ Time completed: _______

Consent Granted for participation: □ Yes □ No

1. **Demographic Information:**
   1. What is the current age of your child? __________years __________ months
   2. When was your child discharged from New Somerset Hospital after they were born dd/mm/yyyy )? ________________________________________________________
   3. What sort of housing do you live in? □ Formal □ Informal dwelling
   4. How many people are living in the home? ________________________________
   5. How many rooms are in the home? □ 1 □ 2 □ 3 □ > 3
   6. Do you have access to water? □ yes, there is a tap inside the house

□ yes, there is a tap outside

□ yes, we have to walk to get water

□ no, we don’t have access to water

- 1. Do you have electricity? □ Yes □ No
  2. What do you use for cooking? □ electricity

□ wood fire

□ gas/paraffin

- 1. Do you have a toilet? □ Yes, inside the home □ Yes, outside □ No
  2. Who is the primary care giver for the child? □ mother

□ father

□ grandmother or grandfather

□ other? Please specify____________

- 1. Information about the mother:
     1. What is the mother’s DOB and age? _________________________________
     2. What is the highest level of education she achieved? ,

□ never attended school □ Grade 7 or less □ Grade 8 or 9

□ Grade 10 □ Grade 12 □ Diploma □ University Degree

- - 1. Is the mother working? □ Yes □ No
    2. If not, is there a reason why not? ___________________________________
  1. Information about the father:
     1. What is the father’s DOB and age? _________________________________
     2. What is the highest level of education he achieved?

□ never attended school □ Grade 7 or less □ Grade 8 or 9

□ Grade 10 □ Grade 12 □ Diploma □ University Degree

- - 1. Is the father working? □ Yes □ No
    2. If not, is there a reason why not? ___________________________________
  1. Do you get a social grant for this child: □ Yes □ No
     1. If so, which social grant? □ CSG (Support grant) □ CDG (Dependency grant)
  2. Are there smokers at home? Including those smoking outside □ Yes □ No

1. **Regarding the Community Health Worker (CHW) visits:**
   1. Were you visited by CHWs after you were discharged from New Somerset Hospital? □ Yes □ No
   2. How many days after your discharge did they visit you?

□ 1 day □ 2 -3 days □ 4-6 days □ 1 week □ > 1 week

- 1. How many times did they visit you in total?

□ Once □ 1-5 times □ 5-10 times □ > 10 times

- 1. What topics did the CHW discuss with you? Please answer Yes or no for each option:
     1. Breastfeeding – the importance, advice on how to breastfeed □ Yes □ No
     2. Danger signs – what to watch for in your child to alert you when to take the child to hospital □ Yes □ No
     3. How to make ORS (oral rehydration solution) □ Yes □ No
     4. Immunisations – the importance of them and where to go to get them □ Yes □ No
     5. Vitamin A and deworming - the importance of it and where to go to get them □ Yes □ No
     6. The Road to health booklet (RTHB) –what is it used of, the importance of bringing it to all hospital or clinic visits, useful information in the RTHC □ Yes □ No
  2. Were there any other topics that were discussed?
  3. Did you find the information helpful? On a scale from 1 to 5 where 5 is extremely helpful and 1 is not helpful at all? Put a circle around your answer

1 2 3 4 5

- 1. What other topics would you find helpful?

1. **Outcomes of child:**
   1. How is you child doing?

□ well □ chronically unwell, if so what is wrong?­­­­­­­­­­­­­­­­_____________________

□ passed away, If so at what age and from what did the child die? __________________________________________________________________

- 1. Do you have RTHB and can they produce it? □ Yes □ No
     1. If no, what is the reason for no RTHB?

□ lost □stolen □ burnt □ at another home □never had one

- 1. Are your child’s Immunisations UTD? CHW to check on the RTHB:

□ Yes □ No □ no RTHB

- 1. What is your child’s most recent weight (rounded up to 1 decimal place)? Either verbally or on RTHB date of it (dd/mm/yyyy)________________________________
  2. What is your child’s most recent length in cm (rounded up to 1 decimal place)? Either verbally or on RTHB date of it (dd/mm/yyyy)___________________________
  3. For how long, in months, did you feed your baby only breast milk and no other liquids including water or solids?

□ < 1 month □ 1month □ 2-3months □ 4-5 months □ 6 months □ > 6 months

- 1. Has the baby ever been admitted to hospital? □ Yes □ No
     1. If your answer is yes, how many times where they admitted?­­­­_____________­­­­­­­
     2. If your answer is yes, why was the baby admitted:

□ diarrhoea □ breathing problems □ infection

□ other:

- 1. Did you ever make contact with the CHW after the visits? □ Yes □ No
     1. If your answer is yes, what was the reason you went to the CHW?
  2. Or if baby ever became sick or you had questions about your child, would you ever contact the CHW for help? □ Yes □ No

1. **In summary:**
   1. Are there any other ways in which the CBS would be helpful for you or other caregivers?

*Thank you for completing the questionnaire!*

**Supplementary file 4:** Flow diagram depicting the referral pathway for VLBW babies

VLBW= very low birth weight; CBS = Community Based Services ; NP = nurse practitioner; CHW = Community Heath Worker NPO = Non-Profit Organisation

**Supplementary file 5:** HCBS referral form analysis (n=44)

|  | Number (n) | Percentage (%) |
| --- | --- | --- |
| **Form sections fully completed:** |  | |
| Patient address | 44 | 100,0% |
| Contact number | 37 | 84,1% |
| Problem list | 41 | 93,2% |
| Medication listed | 42 | 95,5% |
| Clear indication for referral selected | 42 | 95,5% |
| **Reasons for referral:** |  | |
| Substance abuse | 1 | 2,3% |
| Newborn care/KMC | 24 | 54,6% |
| Breastfeeding/nutrition support | 39 | 88,6% |
| Immunisation | 17 | 38,6% |
| Growth problems | 15 | 34,1% |
| Social support | 2 | 4,6% |
| Treatment adherence | 1 | 2,3% |
| Mental health | 1 | 2,3% |
| **Forms signed by caregiver** | 39 | 88,6% |
| **Pre-filled forms used** | 33 | 75,0% |

HCBS = home and community-based services, KMC = kangaroo mother care

**Supplementary file 6:** Demographic characteristics of the study sub-cohort involved in the interviews

|  | Age child at interview (years, months) | Maternal age (years) | Maternal highest LOE | Housing type | Electricity | Water | Paternal involvement | Paternal employment |
| --- | --- | --- | --- | --- | --- | --- | --- | --- |
| Mother 1 | 2yr 10 mo. | 37 | Diploma | Formal | Yes | Piped inside house | Involved and lives with family | Employed |
| Mother 2 | 2 yr. 4 mo. | 34 | Grade 12 | Formal | Yes | Piped inside house | Involved and lives with family | Employed |
| Mother 3 | 2 yr. 3 mo. | 35 | Grade 12 | Informal | Yes | Piped outside house | Involved and lives with family | Unemployed |
| Mother 4 | Demised 9mo old | 31 | Grade 12 | Formal | Yes | Piped inside house | Involved but not living with family | Unemployed |
| Mother 5 | 2 yr. 4 mo. | 36 | College degree | Formal | Yes | Piped inside house | Not involved and not living with family | Employed |

LOE: Level of education
